# Genetic testing for familial hypercholesterolemia in a Finnish cohort of patients with premature coronary artery disease and elevated LDL-C levels

**DOI:** 10.3389/fcvm.2024.1433042

**Published:** 2024-07-26

**Authors:** Antti Jokiniitty, Markku Eskola, Saara Metso, Martin Bogsrud, Heini Huhtala, Tanja Saarela

**Affiliations:** ^1^ Faculty of Medicine and Health Technology, Tampere University, Tampere, Finland; ^2^ Department of Internal Medicine, Tampere University Hospital, Tampere, Finland; ^3^ Heart Hospital, Tampere University Hospital, Tampere, Finland; ^4^ Unit for Cardiac and Cardiovascular Genetics, Department of Medical Genetics, Oslo University Hospital, Oslo, Norway; ^5^ Faculty of Social Sciences, Tampere University, Tampere, Finland; ^6^ Department of Clinical Genetics, Kuopio University Hospital, Wellbeing Services County of North Savo, Kuopio, Finland

**Keywords:** familial hypercholesterolemia, genetic testing, coronary artery disease, screening, hyperlipidemia

## Abstract

**Background:**

Based on Finnish *LDLR*-founder variations, the prevalence of familial hypercholesterolemia (FH) in Finland is estimated to be at least 1:600. Patients with FH have increased risk of premature coronary artery disease (CAD) and thus the prevalence of FH is expected to be higher in this subgroup.

**Objective:**

To assess the prevalence of monogenic FH in a Finnish cohort of patients with premature CAD and elevated low-density lipoprotein cholesterol (LDL-C) levels.

**Methods:**

Among 28,295 patients undergoing angiography at Heart Hospital at Tampere University Hospital between 2007 and 2017, we identified 162 patients diagnosed with premature CAD (men aged <55 years and women aged <60 years) and history of high LDL-C (≥5 mmol/L) levels without secondary causes of hypercholesterolemia. Clinical probability of FH was estimated, and genetic testing of FH was carried out in 80 patients with informed consent.

**Results:**

Of the 80 patients with premature CAD and history of high LDL-C levels, 70% were men; the age at diagnosis of CAD for male and female patients was 48 and 53 years, respectively. In total, 58 (73%) patients had probable (*n* = 54) or definite (*n* = 4) FH based on Dutch Lipid Clinic Network criteria. A pathogenic variant of FH was found in five (6%) patients. Prevalence of the genetically verified FH was 1:16. The FH variant was found in 75% of patients with definite FH.

**Conclusions:**

The prevalence of genetically verified FH was 1:16 among patients with premature CAD and elevated LDL-C level, which is 38 times higher than the estimated prevalence of 1:600 in the general Finnish population.

## Introduction

Monogenic familial hypercholesterolemia (FH) is predominantly caused by autosomal dominant variants of *LDLR*, *APOB*, or *PCSK9* genes that disrupt the normal LDL uptake (1). FH is a common genetic disease, and based on large meta-analyses, the prevalence of heterozygous FH (HeFH) is 1:311–1:313 in the general population (2, 3). Untreated FH leads to accelerated cardiovascular disease (CVD) and premature coronary artery disease (CAD) (4).

FH can be diagnosed with clinical criteria or by molecular genetic testing. Clinical criteria are based on low-density lipoprotein cholesterol (LDL-C) levels, presence of premature CVD, family history of CVD and/or hypercholesterolemia, and clinical findings of lipid accumulation (5, 6). Sensitivity of the clinical criteria can decrease if information regarding family history of CVD and pre-treatment cholesterol levels of affected relatives is missing or suboptimal. In addition, there is marked overlap in phenotypes of polygenic FH with complex molecular etiology and HeFH (7). However, patients with monogenic FH have a two- to threefold increased risk for CAD compared with those with similar LDL-C levels without a monogenic cause (8). A genetic diagnosis of FH enhances cascade testing in the family (7), improves initiation and adherence of lipid-lowering therapies (9), and provides more accurate CVD risk stratification without markedly increased anxiety or mental burden (10). In some areas, a definite FH diagnosis can also affect medical reimbursements. Thus, identifying the pathogenic variant of FH via genetic testing is regarded as the gold standard for diagnosing FH.

Current European Society of Cardiology (ESC) guidelines recommend that a diagnosis of FH should be made using clinical criteria and confirmed with DNA analysis when possible (4). Journal of the American College of Cardiology (JACC) consensus statement recommends FH genetic testing to be standard of care for patients with definite or probable FH and their relatives. The genetic testing should include *LDLR*, *APOB*, and *PCSK9* genes (7)

The Finns present a genetically isolated population. It has previously been reported that five LDLR gene founder variants account for up to 78% of HeFH cases and seven founder variants up to 90% of HeFH cases (11, 12). Based on Finnish *LDLR*-founder variants, the prevalence of FH in Finland is estimated to be at least 1:600 (13). In national current care guidelines, genetic testing for the four most common Finnish founder variants is recommended if FH is definite or probable based on Dutch Lipid Clinic Network (DLCN) criteria. Additional genetic testing is recommended in patients with a strong suspicion of FH and negative founder variant test.

The positive predictive value of DLCN criteria for FH-causing variants is high. In patients with DLCN criteria, the variant detection rates are 54%, 39%, and 28% for definite FH (>8 points), probable FH (6–8 points), and possible FH (3–5 points), respectively (14).

The aim of the present study was to investigate the prevalence of clinical and genetically verified FH in a Finnish cohort of patients with premature CAD and elevated LDL-C levels.

## Methods

We recently developed an automated FH-screening tool implemented at Tampere Heart Hospital (15). We retrospectively identified patients treated between 2007 and 2017 with premature CAD and history of elevated LDL-C levels (≥5 mmol/L) for further evaluation regarding FH (15). In brief, among 28,295 patients undergoing coronary angiography, 211 had premature CAD (men aged < 55 years and women aged < 60 years) and their highest measured LDL-C was ≥5 mmol/L. After extensive analysis of electronic health records (EHR) to exclude patients with secondary hypercholesterolemia, i.e., nephrotic syndrome or end-stage renal disease (ESRD), uncontrolled diabetes mellitus as defined by HbA1c above 70 mmol/L, hypothyroidism (TSH > 10 mU/L), medications (i.e., aripiprazole, anabolic steroids), or cholestasis, 162 patients were candidates for further evaluation regarding FH.

We used the DLCN criteria (16) to evaluate clinical FH in the cohort selected for further evaluation. As done in previous studies, patients with >8 points were considered to have definite FH, those with 6–8 points probable FH, and those with 5 points possible FH. Due to the selection criteria for the screening program, the lowest possible result for participants was 5 points and referral to genetic testing did not depend on the DLCN score in the selected cohort (17).

Genomic DNA was extracted from venous blood samples using standard procedures. Due to the retrospective nature of the study, genetic testing was conducted in two different phases: either during standard treatment, as instructed in national Current Care Guidelines when referral to genetic testing is based on treating clinicians’ awareness or after recruitment in screened patients who were initially not thoroughly evaluated regarding FH.

The assessment of the Finnish FH founder variants (*LDLR*) was conducted in 21 patients and a FH Gene Panel was conducted in three patients as a follow-up during standard clinical surveillance and treatment (15). In the second phase, to enhance genetic testing of FH, we recruited participants who were not studied with a FH Gene Panel (*n* = 159) for further DNA analysis. Patients were studied for Finnish FH founder variants, if not done previously. In addition, FH variants were tested in consenting patients at the Unit for Cardiac and Cardiovascular Genetics, Department of Medical Genetics, Oslo University Hospital.

A genetic analysis for four Finnish founder mutations of the *LDLR* gene (FH-Helsinki, FH-North Karelia, FH-Pori, FH-Turku) was conducted by FimLab. Samples were extracted from peripheral blood (minimum 1 ml) in an ethylenediaminetetraacetic acid (EDTA) tube. Quantitative polymerase chain reaction with TaqMan® SNP Genotyping assay, Applied Biosystems® with specific primers, and probes for each founder mutation was conducted with LightCycler 480 II (Roche®) (Supplementary Table S3A)

A FH Gene Panel during standard treatments was conducted by a Blueprint Genetics® Hyperlipidemia panel using next-generation sequencing (NGS, including sequencing and deletion/duplication analysis). Blueprint Genetics Laboratory located in Finland is a CLIA-certified laboratory and accredited by the College of American Pathologists and by FINAS Finnish Accreditation Service. Samples were extracted from peripheral blood (minimum 1 ml) in an EDTA tube. The target region for each gene includes coding exons and ±20 bp from the exon-intron boundary. Panel content is described in Supplementary Tables S3B and S3C.

Oslo University Hospital conducted Sanger sequencing of the translated exons of the LDLR gene (NM_000527.4) with at least 20 bp of flanking intron sequences, and a 101 bp fragment spanning nucleotides c.10537–10637 in exon 26 of the APOB gene (NM_000384.3) to detect mutations affecting codon 3527, which is the only codon where mutations definitely may cause FDB. Sanger sequencing of the translated exons with flanking intron sequences of the PCSK9 gene (NM_174936.4), as well as multiplex ligation-dependent probe amplification (MLPA) analysis of the LDLR gene, was also performed (18).

In the second phase, a letter explaining the details of the study was mailed to the patients between October 2019 and May 2020 if the patient was still alive and lived in Pirkanmaa Hospital district. The participants agreeing to participate were asked to return their written consent forms to the study and to the genetic testing via mail free of charge.

**Figure 1:**
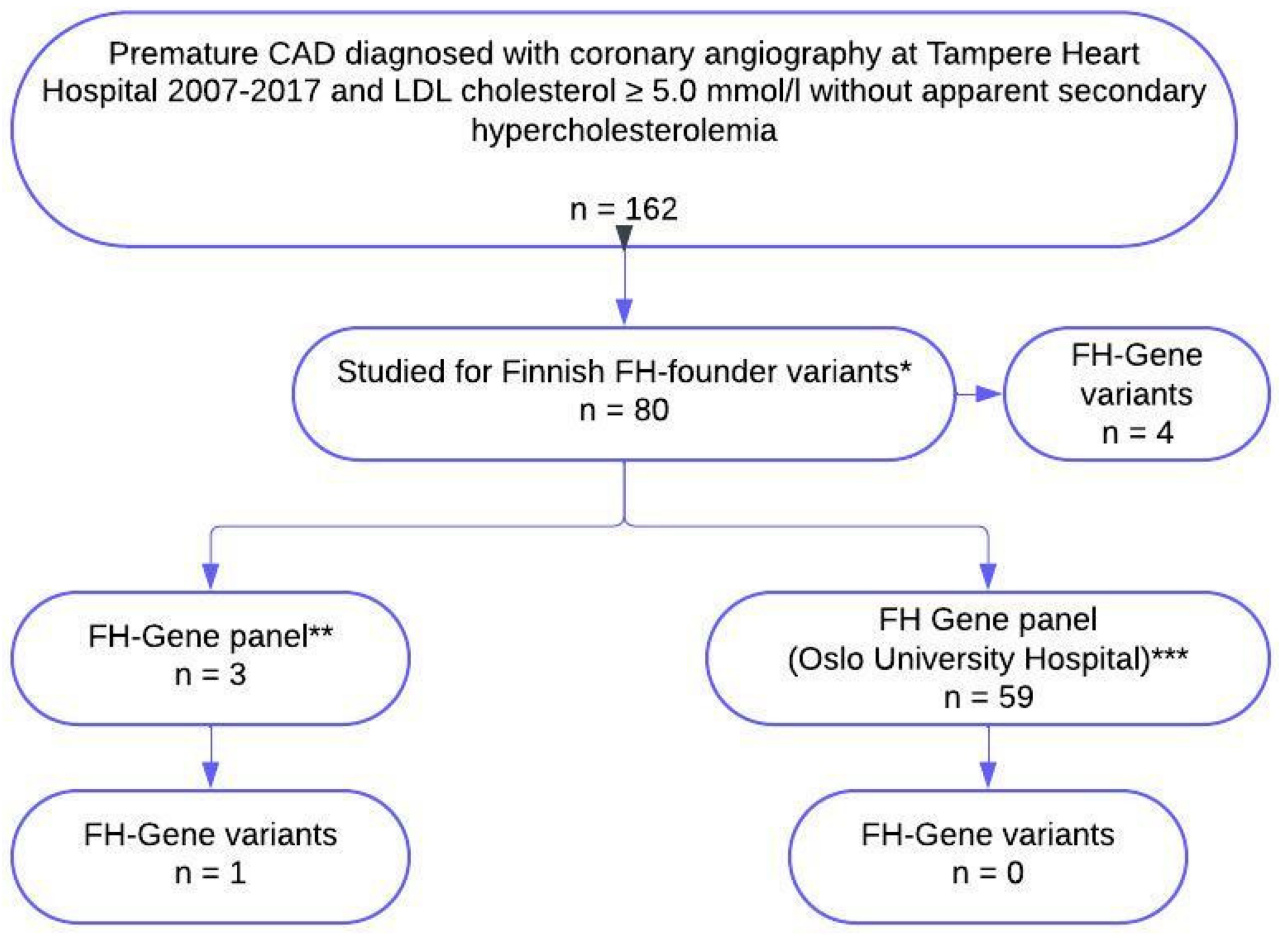
Flow chart of the study. *Four Finnish founder mutations, LDLR (g.39215_47749del8535), LDLR [c.925_931delCCCATCA, p.(Pro309Lysfs)], LDLR [c.1202T>A, p.(Leu401His)], and LDLR [c.2531G>A, p.(Gly844Asp)] in 21 patients were studied during standard treatment and 59 patients were recruited after further evaluation regarding FH. **FH gene panel, NGS (including sequencing and deletion/duplication analysis) including LDLR, APOB, PCSK9, and LDLRRAP1. All three patients were studied during standard treatment. ***Sanger sequencing of the translated exons of the LDLR gene (NM_000527.4) with at least 20 bp of flanking intron sequences, and a 101 bp fragment spanning nucleotides c.10537-10637 in exon 26 of the APOB gene (NM_000384.3) to detect mutations affecting codon 3527, which is the only codon where mutations definitely may cause familial defective apolipoprotein B-100 (FDB). Sanger sequencing of the translated exons with flanking intron sequences of the PCSK9 gene (NM_174936.4), as well as MLPA analysis of the LDLR gene, is also performed. All 59 patients were recruited to genetic testing after further evaluation regarding FH. FH, familial hypercholesterolaemia; CAD, coronary artery disease.

In total, 104 consent letters were sent and 66 (53%) patients agreed to participate in the study and signed the informed consent for genetic testing. Seven patients did not provide blood samples despite their initial consent. Blood samples from 59 patients were collected for genetic testing in the second phase. A flow chart of the study is presented in Figure 1.

To assess the cardiovascular risk profile, diabetes was defined if a patient had a previous diagnosis of diabetes, had received treatment for diabetes, or had an Hba1c level above 48 mmol/mol. Hypertension was defined as systolic blood pressure (BP) >140 mmHg, diastolic BP >90 mmHg, or a diagnosis of hypertension or antihypertensive medication. Smoking was categorized as non-smoker, ex-smoker, or current smoker. Obesity was defined as a body mass index (BMI) >25 kg/m^2^.

The statistical analyses were performed using the SPSS version 26.0 (IBM Corp., Armonk, NY, USA). Absolute numbers and percentages were used to describe the categorical data. Categorical variables were analyzed using a chi-square test. Quantitative data on age and BMI were given as means and standard deviations (SD) and total cholesterol (TC) and LDL-C levels as medians and quartiles. A Mann–Whitney *U*-test was used to assess the difference in continuous variables between the two patient groups. A two-sided *p*-value <0.05 was considered statistically significant.

The study protocol conforms to the ethical guidelines of the 1975 Declaration of Helsinki and the study protocol was previously approved by the ethics committee of Tampere University Hospital.

## Results

Clinical data and the results from genetic testing were available for 80 patients (Figure 1). Of the 80 patients with premature CAD and history of high LDL-C levels, 70% were male. The mean age at the time of CAD was 48.1 years (SD 4.7) in men and 53.2 years (SD 5.5) in women (Table 1). The highest median recorded total cholesterol was 7.3 mmol/L (Q1–Q3: 6.9–7.8 mmol/L) and the highest median LDL-C was 5.4 mmol/L (Q1–Q3: 5.2–5.8 mmol/L). Hypertension was present in 76% of patients, diabetes in 16%, and a family history of early CAD in 70% of patients. Current smoking was reported by 30% of patients and ex-smoking by 35% of patients at the time of CAD. Lipid-lowering therapy was prescribed to 46% of patients before the CAD diagnosis and 70% of these were currently using the prescribed medication (Table 1).

**Table 1:** Clinical characteristics of patients tested for genetic variants of FH (*n* = 80).

Gender, *n* (%)	
Male	56 (70.0)
Female	24 (30.0)
Age at diagnosis of CAD, mean (SD)	
Male	48.1 (4.7)
Female	53.2 (5.5)
TC (mmol/L), median (Q1–Q3)	7.3 (6.9–7.8)
LDL-C (mmol/L), median (Q1–Q3)	5.4 (5.2–5.8)
Family history of early CAD, *n* (%)	56 (70.0)
Hypertension, *n* (%)	61 (76.3)
Diabetes, *n* (%)	13 (16.3)
Current/ex-smoker at diagnosis of CAD, *n* (%)	52 (65)
BMI, mean (SD)	29.5 (5.8)
DLCNC, *n* (%)	
Possible FH	22 (27.5)
Probable FH	54 (67.5)
Definite FH	4 (5)

DLCNC, Dutch Lipid Clinic Network Criteria.

Four patients (5%) were classified with definite FH based on DLCN criteria, 54 (67.5%) patients with probable FH, and 22 (27.5%) patients with possible FH (Table 1 and Supplementary Material).

A heterozygous FH variant was found in five (6%) patients, of which four variants were Finnish founder variants [three FH-North Karelia *LDLR* c.925_931delCCCATCA, p.(Pro309Lysfs) and one FH-Pori *LDLR* c.1202T>A, p.(Leu401His)] and one was LDLR c.(2,140 + 1_2141-1)_(2,548+?)del/Exons 16–18 variant (Table 2 and Supplementary Material). Thus, the prevalence of the genetically verified FH was 1:16 in the study cohort. Among the five patients with genetically confirmed FH, three (60%) fulfilled the DLCN criteria for definite FH and two (40%) for possible FH.

**Table 2:** Genetic variants of familial hypercholesterolemia in the study population.

Gender	Age at diagnosis of CAD	Highest LDL-C (mmol/L)	Family history of early CVD	DLCNC	Gene	Pathogenic (P)/likely pathogenic (LP)
Male	38	8.5	Yes	11	c.925_931delCCCATCA p.(Pro309Lysfs) (FH—North Karelia)	P
Female	42	9	No	10	c.925_931delCCCATCA p.(Pro309Lysfs) (FH-North Karelia)	P
Female	51	7.5	Yes	9	c.(2140+1_2141-1)_(2,548+?)del/Exons 16-18	LP
Female	50	7.4	Yes	8	c.1202T>A p.(Leu401His) (FH-Pori)	P
Female	58	5.4	Yes	6	c.925_931delCCCATCA p.(Pro309Lysfs) (FH—North Karelia)	P

DLCNC, Dutch Lipid Clinic Network Criteria.

The FH variant was found in 75% of those four patients with definite FH compared to only 4% in those 54 patients with possible FH. The five patients with genetically confirmed FH had higher LDL-cholesterol (7.5 vs. 5.5 mmol/L, *p* = 0.001) and were younger (47.8 vs. 49.8 years, *p* = 0.001) than those with a negative genetic test (Table 3). The four founder variants and one additional FH variant in gene panel had been discovered during standard clinical genetic testing as a part of standard treatment.

**Table 3:** Baseline values of patients tested for genetic variants of familial hypercholesterolemia.

	No genetic variant of FH *n* = 75	Genetic variant of FH identified *n* = 5	*p*-value
Age at baseline CAD, mean (SD)	49.8 (5.3)	47.8 (7.9)	0.001
TC (mmol/L), median (Q1–Q3)	7.2 (6.9–7.7)	9.9 (8.2–10.6)	0.010
LDL-C (mmol/L), median (Q1–Q3)	5.3 (5.1–5.7)	7.5 (6.4–8.6)	0.001

## Discussion

In our study the prevalence of FH among young CAD patients who underwent genetic analysis of FH was 6% (1:16), which is 38 times higher than the estimated prevalence of 1:600 in the general Finnish population. This is in line with other reports and is expected due to the high risk of CAD in FH. The prevalence of genetic variants of FH in patients with possible FH based on DLCN criteria was only 4% and based on previous studies is lower than expected.

Based on a large meta-analysis, approximately 1 in 300 individuals is affected by HeFH (2, 3). It appears though that there is a large variability in the prevalence of FH in different regions of the world. In certain founder populations, a prevalence as high as 1:80 has been reported in contrast to the prevalence of 1:836 reported in a recent study from Iceland (19, 20). In the large study by Björnsson et al., clinical FH (based on modified DLCN criteria) was present in 2.2% of the whole study population, of whom monogenic FH was found in 5.2% (20). A previous Finnish study showed a prevalence of 9% of genetically confirmed HeFH in young (aged ≤45 years) coronary heart disease (CHD) patients (21). In the present study, the prevalence of clinical definite or probable FH was 73% (1:1.4) but genetically verified monogenic FH was found in 6% (1:16) among premature CAD patients.

An optimal screening strategy for FH is yet to be established but the selective screening of pathogenic gene variants of FH in patients with premature CAD appears to be an effective strategy in identifying index patients with monogenic FH and provide a possibility for cascade screening, which has been shown to be cost-effective (22).

FH is a complex clinical genetic disease (i.e., over 2,000 reported *LDLR* variants; 32 in *APOB*, 23 in *PCSK9*; 1 in *APOE*, and 4 in *STAP1*) with wide genetic heterogeneity and large phenotypic variations (23). For example, some so-called null variants in the *LDLR* gene cause a decrease in LDL-receptor activity <2% of normal and in defective variants LDL-receptor activity remains in the range of 2%–70% (24). A considerable number of rare FH variants are of uncertain significance. Still, in many patients with the FH phenotype, no causative variants can be detected (25). These challenges must be considered when allocating resources to wider genetic screening. A genetic diagnosis of FH has been shown to provide additional value to treatment, cascade screening, and risk stratification of patients, and it is recommended, if available, for confirmation of clinical diagnosis. Better availability of NGS with increasingly lower costs should further assist clinicians in this task (26).

Patients who did not have a FH mutation in our study may still have polygenic forms of hypercholesterolemia caused by multiple genetic factors resulting in elevated LDL-C levels and risk of early development of atherosclerotic cardiovascular disease (ASCVDs). Polygenic hypercholesterolemia was not evaluated in our study as an international consensus on alleles and loci that should be included in genetic risk scores is lacking.

As shown in the study by Björnsson et al. (20) and in our study, hypercholesterolemia in both monogenic FH and complex hypercholesterolemia is markedly undertreated. In addition to hypercholesterolemia, other known cardiovascular risk factors were recorded in the vast majority of the patients in our study. Better screening, identification, and treatment of patients with other well-known risk factors, such as positive family history for CVD, hypertension, smoking, diabetes, high triglycerides, low high-density lipoprotein cholesterol (HDL-C) and high lipoprotein(a), are warranted.

A major limitation of the current study is that there is a possibility of selection bias since 50% of the clinically probable, definite, or possible FH patients in the screened population did not undergo genetic testing for FH variants because of the lack of consent to the study or premature death, other severe disease, or moving out of Pirkanmaa Hospital district. It is possible that some gene variants would have been discovered in these patients. In addition, the prevalence of FH in patients with premature CAD in our study needs to be interpreted with caution as it is solely based on patients tested for genetic mutations. An automated screening tool for FH used in this study identifies patients based on the presence of premature CAD and elevated LDL-C levels. In its current state, the screening tool is unable to calculate pre-treatment levels of LDL-C thus creating a possibility that patients with pre-treatment LDL-C values above 5.0 mmol/L might be excluded by the screening tool if studied in a different laboratory.

Only three patients were tested for the rare genetic causes of FH, i.e., autosomal recessive pathogenic variants of *LDLRAP1* (27), autosomal dominant *APOB* variants (28), and *APOE* (29) variants. Thus, it is possible that they could have been missed in this study.

In conclusion, the prevalence of clinically probable or definite FH was 1:1.4 while the genetically verified FH was 1:16 among patients with premature CAD and elevated LDL-C levels who underwent genetic analysis. Since the prevalence of the genetically verified FH was 38 times higher than the estimated prevalence of 1:600 in the general Finnish population, our results suggest that the screening of high-risk patients for FH with genetic testing is rational.

## Data Availability

The datasets presented in this study can be found in online repositories. The names of the repository/repositories and accession number(s) can be found in the article/Supplementary Material.
