# Supplementary material for: Genetic testing for familial hypercholesterolemia in a Finnish cohort of patients with premature coronary artery disease and elevated LDL-C levels
Source: Front Cardiovasc Med. 2024 Jul 26;11:1433042. doi: 10.3389/fcvm.2024.1433042 (PMC11310056; doi:10.3389/fcvm.2024.1433042)
Supplement: Supplementary file 1 [file Table1.docx]

**Supplementary Material**

Manuscript:

**Genetic testing for familial hypercholesterolemia in a Finnish cohort of patients with premature coronary artery disease and elevated LDL-C levels**

**.** Jokiniitty et al.

**Supplementary table 1**. Dutch Lipid Clinic Network diagnostic criteria for Familial Hypercholesterolemia ^1-2^

| Criteria | Points |
| --- | --- |
| **Family history** |  |
| First-degree relative with known premature* coronary and vascular disease, OR 1 First-degree relative with known LDL-C level above the 95th percentile | 1 |
| First-degree relative with tendinous xanthomata and/or arcus cornealis, OR 2 Children aged less than 18 years with LDL-C level above the 95th percentile | 2 |
| **Clinical history** |  |
| Patient with premature* coronary artery disease | 2 |
| Patient with premature* cerebral or peripheral vascular disease | 1 |
| **Physical examination** |  |
| Tendinous xanthomata | 6 |
| Arcus cornealis prior to age 45 years | 4 |
| **Cholesterol levels mmol/liter (mg/dl)** |  |
| LDL-C ≥ 8.5 (330) | 8 |
| LDL-C 6.5–8.4 (250 – 329) | 5 |
| LDL-C 5.0–6.4 (190 – 249) | 3 |
| LDL-C 4.0–4.9 (155 – 189) | 1 |
| **DNA analysis**** |  |
| Functional mutation in the LDLR, apo B or PCSK9 gene | 8 |
| **Diagnosis (diagnosis is based on the total number of points obtained)** |  |
| Definite Familial Hypercholesterolemia | ≥9 |
| Probable Familial Hypercholesterolemia | 6 – 8 |
| Possible Familial Hypercholesterolemia | 3 – 5 |
| Unlikely Familial Hypercholesterolemia | <3 |

* Premature = < 55 years in men; < 60 years in women

LDL-C = low density lipoprotein cholesterol; FH, familial hypercholesterolemia.

LDLR = low density lipoprotein receptor

Apo B = apolipoprotein B

PCSK9 = Proprotein convertase subtilisin/kexin type 9

**Supplementary table 2. Individual list of patients studied for genetic mutations of FH**

|  | | | | | | | | | |
| --- | --- | --- | --- | --- | --- | --- | --- | --- | --- |
|  | |  |  |  |  |  |  |  |  |
|  | | Age at baseline CVE | Highest LDLC (mmol/l) | Family history of premature CVD | DLCNC score | Finnish Founder mutations | FH Gene Panel (BP – Genetics) | Oslo University FH - Gene panel | Genetic finding |
| Female | 1 | 46 | 9 | Yes | 12 | x |  |  |  |
| Female | 2 | 42 | 9 | No | 10 | x |  |  | FH – North - Karelia |
| Female | 3 | 51 | 7.5 | Yes | 9 | x | x |  | c.(2140+1_21411)_(2548+?)del |
| Female | 4 | 50 | 7.4 | Yes | 8 | x |  |  | FH – Pori |
| Female | 5 | 53 | 6.1 | Yes | 6 | x |  |  |  |
| Female | 6 | 58 | 5.4 | Yes | 6 | x |  |  | FH – North Karelia - |
| Female | 7 | 47 | 5 | Yes | 6 | x | x |  |  |
| Female | 8 | 59 | 5 | Yes | 6 | x |  | x |  |
| Female | 9 | 57 | 5.2 | Yes | 6 | x |  | x |  |
| Female | 10 | 42 | 5.2 | Yes | 6 | x |  | x |  |
| Female | 11 | 52 | 6 | Yes | 6 | x |  | x |  |
| Female | 12 | 59 | 5.8 | Yes | 6 | x |  | x |  |
| Female | 13 | 50 | 5.8 | Yes | 6 | x |  | x |  |
| Female | 14 | 49 | 5.5 | Yes | 6 | x |  | x |  |
| Female | 15 | 59 | 5.4 | Yes | 6 | x |  | x |  |
| Female | 16 | 53 | 5.3 | Yes | 6 | x |  | x |  |
| Female | 17 | 57 | 5.1 | Yes | 6 | x |  | x |  |
| Female | 18 | 58 | 5.1 | Yes | 6 | x |  | x |  |
| Female | 19 | 60 | 5.2 | No | 5 | x |  |  |  |
| Female | 20 | 57 | 5.1 | No | 5 | x |  |  |  |
| Female | 21 | 51 | 5.6 | No | 5 | x |  | x |  |
| Female | 22 | 59 | 5.2 | No | 5 | x |  | x |  |
| Female | 23 | 50 | 5.2 | No | 5 | x |  | x |  |
| Female | 24 | 59 | 5 | No | 5 | x |  | x |  |
|  |  |  |  |  |  |  |  |  |  |
| Male | 1 | 38 | 8.5 | Yes | 11 | x |  |  | FH – North Karelia |
| Male | 2 | 47 | 6.6 | Yes | 8 | x |  |  |  |
| Male | 3 | 47 | 6.5 | Yes | 8 | x |  | x |  |
| Male | 4 | 44 | 7.3 | Yes | 8 | x |  | x |  |
| Male | 5 | 49 | 6.6 | Yes | 8 | x |  | x |  |
| Male | 6 | 40 | 5 | Yes | 7 | x |  | x |  |
| Male | 7 | 53 | 6.6 | No | 7 | x |  | x |  |
| Male | 8 | 44 | 5.6 | Yes | 6 | x |  |  |  |
| Male | 9 | 42 | 5.2 | Yes | 6 | x |  |  |  |
| Male | 10 | 39 | 5.5 | Yes | 6 | x |  |  |  |
| Male | 11 | 48 | 5.9 | Yes | 6 | x |  |  |  |
| Male | 12 | 45 | 5.4 | Yes | 6 | x | x |  |  |
| Male | 13 | 48 | 5.3 | Yes | 6 | x |  | x |  |
| Male | 14 | 50 | 5 | No | 6 | x |  | x |  |
| Male | 15 | 48 | 5.1 | No | 6 | x |  | x |  |
| Male | 16 | 50 | 5.2 | Yes | 6 | x |  | x |  |
| Male | 17 | 46 | 5.1 | Yes | 6 | x |  | x |  |
| Male | 18 | 49 | 5.2 | No | 6 | x |  | x |  |
| Male | 19 | 54 | 5.1 | Yes | 6 | x |  | x |  |
| Male | 20 | 48 | 5.2 | Yes | 6 | x |  | x |  |
| Male | 21 | 52 | 5.2 | Yes | 6 | x |  | x |  |
| Male | 22 | 49 | 5.4 | No | 6 | x |  | x |  |
| Male | 23 | 52 | 6.4 | Yes | 6 | x |  | x |  |
| Male | 24 | 49 | 6 | Yes | 6 | x |  | x |  |
| Male | 25 | 50 | 5.3 | Yes | 6 | x |  | x |  |
| Male | 26 | 48 | 5.8 | Yes | 6 | x |  | x |  |
| Male | 27 | 53 | 5.8 | Yes | 6 | x |  | x |  |
| Male | 28 | 49 | 5.8 | Yes | 6 | x |  | x |  |
| Male | 29 | 50 | 5.7 | Yes | 6 | x |  | x |  |
| Male | 30 | 52 | 5.7 | Yes | 6 | x |  | x |  |
| Male | 31 | 53 | 5.2 | Yes | 6 | x |  | x |  |
| Male | 32 | 49 | 5.1 | Yes | 6 | x |  | x |  |
| Male | 33 | 48 | 5.3 | Yes | 6 | x |  | x |  |
| Male | 34 | 44 | 5.3 | Yes | 6 | x |  | x |  |
| Male | 35 | 48 | 5.1 | Yes | 6 | x |  | x |  |
| Male | 36 | 47 | 5.2 | Yes | 6 | x |  | x |  |
| Male | 37 | 51 | 5.1 | Yes | 6 | x |  | x |  |
| Male | 38 | 51 | 5.1 | Yes | 6 | x |  | x |  |
| Male | 39 | 35 | 5.1 | Yes | 6 | x |  | x |  |
| Male | 40 | 43 | 5 | Yes | 6 | x |  | x |  |
| Male | 41 | 51 | 6.1 | No | 5 | x |  |  |  |
| Male | 42 | 55 | 6.2 | No | 5 | x |  |  |  |
| Male | 43 | 44 | 5.6 | No | 5 | x |  |  |  |
| Male | 44 | 54 | 5.6 | No | 5 | x |  |  |  |
| Male | 45 | 44 | 5.3 | No | 5 | x |  |  |  |
| Male | 46 | 52 | 5.7 | No | 5 | x |  | x |  |
| Male | 47 | 54 | 5.7 | No | 5 | x |  | x |  |
| Male | 48 | 54 | 5.5 | No | 5 | x |  | x |  |
| Male | 49 | 54 | 5.5 | No | 5 | x |  | x |  |
| Male | 50 | 45 | 5.5 | No | 5 | x |  | x |  |
| Male | 51 | 47 | 5.4 | No | 5 | x |  | x |  |
| Male | 52 | 48 | 5.3 | No | 5 | x |  | x |  |
| Male | 53 | 48 | 5.2 | No | 5 | x |  | x |  |
| Male | 54 | 36 | 5.2 | No | 5 | x |  | x |  |
| Male | 55 | 54 | 5.1 | No | 5 | x |  | x |  |
| Male | 56 | 54 | 5 | No | 5 | x |  | x |  |

Supplementary table 3a - Finnish founder variants

| Gene | Genomic location HG19 | HGVS | RefSeq | RS-number | Name |
| --- | --- | --- | --- | --- | --- |
| *LDLR* | chr19: 11123590-11132124 | g.39215_47749del8535 | NG_009060.1 |  | FH-Helsinki |
| *LDLR* | chr19:11107498-11107505 | c.925_931delCCCATCA  p.(Pro309Lysfs) | NM_000527.5 | 387906304 | FH-North-Karelia |
| *LDLR* | chr19:11113293 | c.1202T>A  p.(Leu401His) | NM_000527.5 | 121908038 | FH-Pori |
| *LDLR* | chr19:11129654 | c.2531G>A  p.(Gly844Asp) | NM_000527.5 | 121908037 | FH-Turku |

## Supplementary Table 3b - Panel Content: Genes in the Hyperlipidemia Panel (Blueprint genetics®)

| HGNC approved gene symbol |
| --- |
| *ABCA1* |
| *ABCG5* |
| *ABCG8* |
| *ALMS1* |
| *APOA1* |
| *APOA5* |
| *APOB* |
| *APOC2* |
| *APOC3* |
| *APOE* |
| *CREB3L3* |
| *GPIHBP1* |
| *LDLR* |
| *LDLRAP1* |
| *LIPA* |
| *LMF1* |
| *LPL* |
| *PCSK9* |

#### Supplementary Table 3c - Non-coding variants covered by Hyperlipidemia Panel (Blueprint Genetics®)

| Gene | Genomic location HG19 | HGVS | RefSeq | RS-number |
| --- | --- | --- | --- | --- |
| *ABCA1* | Chr9:107549295 | c.6205-39delT | NM_005502.3 | rs572405590 |
| *ABCA1* | Chr9:107567035 | c.4465-34A>G | NM_005502.3 |  |
| *ABCA1* | Chr9:107571856 | c.4176-11T>G | NM_005502.3 |  |
| *ABCA1* | Chr9:107599404 | c.1195-27G>A | NM_005502.3 | rs200563809 |
| *ABCA1* | Chr9:107690213 | c.-93+2dupT | NM_005502.3 |  |
| *APOA1* | Chr11:116708299 | c.-21+22G>A | NM_000039.1 |  |
| *APOA1* | Chr11:116708365 | c.-65A>C | NM_000039.1 |  |
| *APOC3* | Chr11:116701284 | c.-13-2A>C | NM_000040.1 |  |
| *LDLR* | Chr19:11199939 |  | NM_000527.4 |  |
| *LDLR* | Chr19:11199958 | c.-267A>G | NM_000527.4 |  |
| *LDLR* | Chr19:11199997 | c.-228G>C | NM_000527.4 | rs376713337 |
| *LDLR* | Chr19:11200000 |  | NM_000527.4 |  |
| *LDLR* | Chr19:11200019 | c.-206C>T | NM_000527.4 | rs549995837 |
| *LDLR* | Chr19:11200031 |  | NM_000527.4 | rs1270618112 |
| *LDLR* | Chr19:11200032 |  | NM_000527.4 | rs879254362 |
| *LDLR* | Chr19:11200032 |  | NM_000527.4 |  |
| *LDLR* | Chr19:11200034 | c.-191C>A | NM_000527.4 |  |
| *LDLR* | Chr19:11200037 | c.-188C>T | NM_000527.4 |  |
| *LDLR* | Chr19:11200038 | c.-185_-183delCTT | NM_000527.4 |  |
| *LDLR* | Chr19:11200053 | c.-172G>A | NM_000527.4 |  |
| *LDLR* | Chr19:11200057 | c.-168A>G | NM_000527.4 |  |
| *LDLR* | Chr19:11200062 | c.-163T>C | NM_000527.4 |  |
| *LDLR* | Chr19:11200064 | c.-161A>C | NM_000527.4 |  |
| *LDLR* | Chr19:11200069 | c.-156C>T | NM_000527.4 |  |
| *LDLR* | Chr19:11200069 | c.-155_-154delACinsTTCTGCAAACTCCT | NM_000527.4 |  |
| *LDLR* | Chr19:11200069 | c.-155_-150delACCCCA | NM_000527.4 |  |
| *LDLR* | Chr19:11200070 | c.-155_-154delACinsTTCTGCAAACTCCT | NM_000527.4 | rs879254365 |
| *LDLR* | Chr19:11200070 | c.-155_-150delACCCCAinsTT | NM_000527.4 |  |
| *LDLR* | Chr19:11200071 | c.-154C>T | NM_000527.4 |  |
| *LDLR* | Chr19:11200072 | c.-153C>T | NM_000527.4 |  |
| *LDLR* | Chr19:11200073 | c.-152C>T | NM_000527.4 |  |
| *LDLR* | Chr19:11200074 | c.-151C>G | NM_000527.4 |  |
| *LDLR* | Chr19:11200075 | c.-150A>G | NM_000527.4 |  |
| *LDLR* | Chr19:11200076 | c.-149C>A | NM_000527.4 |  |
| *LDLR* | Chr19:11200079 | c.-146C>A | NM_000527.4 |  |
| *LDLR* | Chr19:11200083 | c.-142C>G/T | NM_000527.4 |  |
| *LDLR* | Chr19:11200084 | c.-139_-130delCTCCCCCTGC | NM_000527.4 |  |
| *LDLR* | Chr19:11200085 | c.-140C>A/G/T | NM_000527.4 | rs875989887 |
| *LDLR* | Chr19:11200086 | c.-139C>A/G | NM_000527.4 |  |
| *LDLR* | Chr19:11200086 | c.-138delT | NM_000527.4 | rs387906307 |
| *LDLR* | Chr19:11200087 | c.-138T>C | NM_000527.4 |  |
| *LDLR* | Chr19:11200088 | c.-137C>T | NM_000527.4 |  |
| *LDLR* | Chr19:11200089 | c.-136C>G/T | NM_000527.4 |  |
| *LDLR* | Chr19:11200089 | c.-136C>G | NM_000527.4 | rs879254374 |
| *LDLR* | Chr19:11200089 | c.-136C>T | NM_000527.4 |  |
| *LDLR* | Chr19:11200090 | c.-135C>G | NM_000527.4 |  |
| *LDLR* | Chr19:11200091 | c.-134C>T | NM_000527.4 |  |
| *LDLR* | Chr19:11200098 | c.-124dupA | NM_000527.4 |  |
| *LDLR* | Chr19:11200105 | c.-120C>T | NM_000527.4 | rs875989886 |
| *LDLR* | Chr19:11200124 | c.-101T>C | NM_000527.4 | rs747068848 |
| *LDLR* | Chr19:11200126 | c.-99A>G | NM_000527.4 |  |
| *LDLR* | Chr19:11200127 | c.-98C>T | NM_000527.4 |  |
| *LDLR* | Chr19:11200202 | c.-23A>C | NM_000527.4 | rs763282380 |
| *LDLR* | Chr19:11200202 | c.-22delC | NM_000527.4 | rs879254379 |
| *LDLR* | Chr19:11200211 | c.-14C>A | NM_000527.4 |  |
| *LDLR* | Chr19:11218203 | c.940+14delC | NM_000527.4 | rs879254730 |
| *LDLR* | Chr19:11221315 | c.941-13T>A | NM_000527.4 |  |
| *LDLR* | Chr19:11224179 | c.1359-31_1359-23delGCGCTGATGinsCGGCT | NM_000527.4 |  |
| *LDLR* | Chr19:11224186 | c.1359-25A>G | NM_000527.4 |  |
| *LDLR* | Chr19:11227685 | c.1845+11C>G | NM_000527.4 |  |
| *LDLR* | Chr19:11227689 | c.1845+15C>A | NM_000527.4 |  |
| *LDLR* | Chr19:11231284 | c.2140+86C>G | NM_000527.4 |  |
| *LDLR* | Chr19:11231301 | c.2140+103G>T | NM_000527.4 |  |
| *LDLR* | Chr19:11242035 | c.*43G>A | NM_000527.4 | rs879254527 |
| *LDLRAP1* | Chr1:25870164 | c.-17_-12dupGGCGGC | NM_015627.2 |  |
| *LDLRAP1* | Chr1:25891056 | c.748-608G>A | NM_015627.2 |  |
| *LPL* | Chr8:19796711 | c.-241G>C | NM_000237.2 | rs540525285 |
| *LPL* | Chr8:19796725 | c.-227T>C | NM_000237.2 |  |
| *PCSK9* | Chr1:55505180 | c.-331C>A | NM_174936.3 | rs778796405 |

References:

1. Nordestgaard BG, Chapman MJ, Humphries SE, et al. Familial hypercholesterolaemia is underdiagnosed and undertreated in the general population: guidance for clinicians to prevent coronary heart disease: consensus statement of the European Atherosclerosis Society. European heart journal. 2013;34:3478-3490a.
2. World Health Organization. Familial hypercholesterolemia—report of a second WHO Consultation. Geneva, Switzerland: World Health Organization. 1999. (WHO publication no. WHO/HGN/FH/CONS/99.2).
